# The Role of Formal Schooling, Literacy, and Health Knowledge in Addressing Domestic Violence Against Women in West Africa

**DOI:** 10.3390/ijerph21111492

**Published:** 2024-11-09

**Authors:** Amelia Van Komen, Hayley Pierce

**Affiliations:** Department of Sociology, School of Family, Home and Social Sciences, Brigham Young University, Provo, UT 84602, USA; amelia.vankomen@gmail.com

**Keywords:** domestic violence, West Africa, education, literacy, health knowledge

## Abstract

When “education” is cited as a solution for domestic violence, different aspects of knowledge acquisition are often omitted. This study uses 2019 Demographic and Health Surveys from four West African countries (The Gambia, Liberia, Senegal, Sierra Leone) with a combined sample size of 12,480 women and generalized ordered logit regression to examine the effects of types of knowledge (years of schooling, literacy, and health knowledge) on domestic violence (physical abuse, emotional abuse, and control issues). The results suggest that literacy has the most reliable beneficial impact on domestic violence and was consistently associated with decreased odds of abuse. However, greater findings suggest that schooling, literacy, and health knowledge function as separate types of education and that their relationships are complex and context-specific. By neglecting to see these types of knowledge as separate entities that can operate together, it is possible that mitigation strategies for domestic violence are going undiscovered.

## 1. Introduction

Domestic violence (it is important to note that while the term domestic violence can sometimes refer to the broader type of violence between any two members of a familial setting rather than just between intimate partners, researchers use the terms domestic violence (DV) and intimate partner violence (IPV) fairly synonymously (World Health Organization, 2013); for the purposes of this paper, the two terms will be used interchangeably, congruent with the intended definition in the discussed articles) (DV) is a widespread form of violence around the world, reaching especially concerning rates for women in Sub-Saharan African countries [1,2]. DV includes physical, sexual, and emotional abuse as well as controlling behaviors by an intimate partner. Researchers use “education” as a common avenue for explaining and working towards mitigating DV rates. However, this research is largely based on the common and easily quantifiable measure of years of schooling. Despite a large breadth of literature on the intersection between education and DV, there are mixed findings on whether becoming educated protects women from violence in their homes. These mixed findings, among reasons such as context, sample, etc., may suggest that “education” means more than attending school, and that researching “education” in this way may not be sufficient [3,4]. Breaking down what it means to be educated and to hold knowledge can help us better understand the experiences individuals face and what types of education impacts issues such as DV.

In Sub-Saharan Africa, DV exceeds the global average (30%) with a prevalence of 36%, with women in Africa being subject to more lifetime partner violence (45.6%) than individuals anywhere else in the world [2]. These high rates are attributed to the historical traditional gender roles and patriarchal values embedded in many Sub-Saharan cultures [5]. These ideals begin in early life, as male children are prioritized, and all children are socialized to customs that perpetuate violence against girls and women. Justification for DV continues through adolescence and early adulthood as traditional outlooks on marriage persist and the average age at marriage for women remains low [6].

These complicated issues and societal beliefs create exceptionally high levels of DV in Sub-Saharan Africa, leading researchers and advocates to search for further causes and solutions. For example, the common conceptual modeling of DV revolves around economic characteristics [7] and attitudes [8] of individuals. A woman’s risk of DV is associated with low socioeconomic status, rural residence, and disempowerment [9,10]. Odds of DV are also higher among working women than non-working women in West Africa, and communities with higher degrees of economic inequality have higher levels of abuse [7].

The most proposed avenue to reduce DV is schooling, despite conflicting findings. Schooling most commonly references attendance in a formal education process where individuals are taught by educators in some form or the completion of primary education. In terms of global DV, research finding schooling to be a protective factor has shown that the odds of recent DV among women without any education can be upwards of 5.61 times that of college-educated women [11] and that, broadly, greater years of schooling are associated with less violence [12,13,14]. However, Erten and Keskin [15] and Cools and Kotsadam [7] explore the idea that formal education through schooling does not always translate to increased empowerment and decreased violence. Their findings showed that female schooling improved women’s economic standings but did not create better empowerment outcomes in the familial setting [15], potentially due to a disruption in status norms [7]. Others found that there are no significant associations with a woman’s formal education and DV [16,17].

Research that identifies years of schooling as a protective factor against DV posits the main functionalities to be altered attitudes, bargaining power, and increased decision making. Rapp et al. [18] found that wives with more years of formal schooling than their husbands experienced less DV compared to equally low-educated spouses and that relationships with the same years of schooling had the lowest likelihood of DV. These systematic changes in the level of schooling likely shift gender dynamics, allowing women the ability to participate in decision making regarding themselves, their families, and their communities [19].

These mixed findings, among reasons such as context, sample, etc., may suggest that “education” means more than attending school and that researching “education” in this way may not be sufficient [3,4]. Other facets of education, such as literacy—conventionally defined as the ability to identify, understand, communicate, and compute using printed and written materials [20]—and health-specific knowledge—referring to the knowledge and ability to acquire, understand, and evaluate health information to make judgments and decisions regarding healthcare [21]—may be helpful in understanding the experiences individuals face and what types of education impacts issues such as DV.

There has been a continued assumption that educational attainment data can be used to estimate adult literacy rates despite emerging evidence that schooling does not translate to reading and writing proficiency [22]. Many women in Africa who have completed several years of primary school cannot read, and, conversely, there are many women in Sub-Saharan Africa who never attend a formal school but hold reading and writing skills [3]. Measuring only years of schooling may ignore the quality of schools and programs, strain on school systems, attendance, and retention of skills [3,23].

Broadly, literacy correlates with lower domestic violence rates [24,25,26]. Like schooling, literacy affects DV through the lens of gender equality and altered attitudes. Literacy can change cultural justifications of wife-beating and other types of DV [27,28]. Regarding community literacy, these impacts on the societal view of women suggest that increasing regional and household levels of literacy could do more to decrease DV than focusing on individual status [25]. Likewise, key findings point to the impact on spousal literacy where urban literate women with a literate spouse are the least likely to have experienced physical violence, whereas rural literate women with an illiterate spouse may have the highest odds [27].

Health-specific knowledge is another facet of education that stands distinct from schooling or literacy. Generally, health knowledge, or health literacy, has been shown to improve women’s lives and health outcomes in developing countries [29], including West African countries, which experience health literacy rates as low as one-third [30]. Health knowledge interventions are suggested to decrease DV through increased empowerment and screening [31,32]. Other schools of thought note that health literacy positively impacts health outcomes only through addressing multiple modes of education [29].

The existing research on the relationship between DV and “education” is nuanced and lacks consensus. Regardless, the typical ways of measuring “education” through formal schooling ignores the complicated and distinct aspects of various forms of education and knowledge acquisition. To better understand possible mitigation strategies for decreasing the rates of DV, more research is needed to explore specific types of education and what aspects of education can help protect women.

This study aims to fill this information gap by treating areas of education as distinct entities—formal years of schooling, reading literacy, and health knowledge—using data from The Gambia, Liberia, Senegal, and Sierra Leone 2019 Demographic Health Surveys (DHS), a nationally representative household survey of women of childbearing age that provides data for monitoring indicators in the areas of health, nutrition, and population. This study’s objective is to examine three distinct types of education (years of formal schooling, literacy, health knowledge) and their corresponding impact on three forms of DV (physical abuse, emotional abuse, and control issues) using generalized ordered logistic regression models.

## 2. Methods

The data come from the DHS, which provides relevant indicators regarding health and population. The DHS program assists in collecting, analyzing, and disseminating data in these areas through more than 400 surveys in over 90 countries. These data have advanced global understanding of women’s empowerment, health, and wellbeing, as well as other indicators, in developing countries. Surveys are administered by the DHS program, funded by the U.S. Agency for International Development and by statistical, developmental, and/or governmental entities in each country. To ensure each survey is nationally representative, surveys are based on national probability sampling. Throughout the survey timeline of eighteen to twenty months, many procedures are followed to guarantee proper questionnaire design, personnel training, distribution, data processing, and reporting. Questions on domestic violence were only asked of a subset of women who were randomly selected and interviewed in privacy for the domestic violence module (for more information about the domestic violence module, please see https://dhsprogram.com/data/Guide-to-DHS-Statistics/17_Domestic_Violence.htm URL accessed on 15 January 2024). Data for this analysis are from the most recent surveys (2019) available in West Africa and include The Gambia, Liberia, Senegal, and Sierra Leone. Countries were selected due to their geographical location of West Africa and the availability of the domestic violence module. Sample sizes for each country are available in Table 1.

We note important limitations to the data used here. This research is limited to the health and empowerment variables included in the DHS survey. Questions are selected by both the DHS and supporting organizations, but the surveys do not touch on every aspect of domestic violence or health knowledge. Also, as the nature of domestic violence is sensitive, there is a chance for under-reporting of the prevalence of violence. Another limitation is the inability to measure and test the institutional differences between the three pathways of knowledge. We noted the population, content, and delivery differences but were unable to empirically measure or test differences in this data set.

### 2.1. Measures

#### 2.1.1. Domestic Violence

We used respondent’s self-reports to determine experiences with domestic partner violence in three domains: physical abuse, emotional abuse, and control issues. Each woman was asked if they had never, often, sometimes, or “yes, but not in the last 12 months” experienced a variety of domestic violence acts. These acts included things such as “being pushed, shook or having had something thrown at them (physical abuse),” “threatened with harm (emotional abuse),” and “husband/partner accuses respondent of unfaithfulness (control issues)” (see Appendix A, Table A1 for a full list of items). We coded each act into a dichotomous variable (0 = never, 1 = any experience) and then created four summed variety scales: physical abuse (Kuder–Richardson reliability = 0.79), emotional abuse (Kuder–Richardson reliability = 0.73), control issues (Kuder–Richardson reliability = 0.78), and one final scale for overall abuse that includes all measures from the previous three scales and additional questions about sexual abuse (Kuder–Richardson reliability = 0.86).

#### 2.1.2. Sources of Knowledge

First, schooling was based on self-reported years of education completed (ranging from 0 to 25 years). Second, literacy was a dichotomous variable comparing women who could read a whole sentence (coded as 1) to women who could not read a whole sentence (coded as 0). The DHS respondents who reported not having “higher than secondary school” education were asked to read a card with a simple sentence in their selected language. As such, this is not a test of reading English or even reading in the dominant national language; rather, it is a test of reading in the language the respondent selected. This classification of literacy does not measure comprehension or other indicators of literacy such as the ability to evaluate, engage with, or use specific written words.

Third, health knowledge was a composite variable composed of six dichotomous items summed together. The six items included access to magazines, access to radio, access to TV, hearing about family planning in the media, knowledge of oral rehydration, and distance to a health facility. These variables were selected from a robust, substantiated indicator of health literacy available in the DHS [33]. The measure relies on an individual’s capacity to interpret, obtain, and understand health information and the ability to make appropriate health decisions. The six variables include what could be utilized by or taught at a health education class, could be discussed between women in a community, or could be learned through day-to-day living. Regarding coding, health knowledge comprised six dichotomous items. A woman’s use of magazines, radio, and TV were each coded as 1 if they used magazines, radio, or TV any time (coded as 1) compared to women that did not use these resources at all (coded as 0). Capacity to hear about family planning was coded as 1 if the respondent reported that they had heard about family planning on the TV, radio, newspaper, and/or text message in the last few months (coded as 1). In addition, knowledge of oral rehydration was coded as 1 if a woman had heard of or used oral rehydration. Lastly, distance to a health facility was coded as 1 if the distance to a woman’s health facility was no problem or not a big problem and 0 if it was a big problem. These items were then added together, such that higher scores indicated higher health knowledge, ranging from 0 to 6.

#### 2.1.3. Control Measures

The measure of participation in decision making included the variables “person who usually decides on respondent’s health care”, “person who usually decides on large household purchases”, and “person who usually decides on visits to family or relatives”. This measure compares respondents who are never involved in decision making (coded as 0) to those involved in one of the variables or more (coded as 1). The number of other wives was coded as 0 for no other wives and coded as 1 for at least one other wife. Justified beating included justifying the beating of a wife for the following scenarios: whether it was justified for a husband to beat his wife if she goes out, if she neglects children, if she argues, if she refuses sex, and if she burns food. This measure compares respondents who never justified beating (coded as 0) to those who justified violence in any scenario (coded as 1).

The additional control variables included wealth, urban residence, marital status, number of children, and age. Wealth is a wealth index factor score. Urban residence is a dichotomous variable (coded 1 = urban and 0 = rural). Marital status compared women who were married (coded as 1) to those who had never been in a union, lived with a partner, were widowed, no longer lived with a partner/husband, or were separated (coded 0). Number of children was the self-reported number of living children and age was the respondent’s current age in years. The husband’s education was reported by the respondent and was the number of years of education completed. Mean replacement was used for instances where the respondent did not know the number of years of education of their partner.

### 2.2. Analytic Strategy

Table 2 presents the descriptive statistics (percent or mean values) for our full sample, and then those parsed out by country. Table 3 shows the relationships between schooling, literacy, and health knowledge and each form of abuse (physical, emotional, control issues, and any abuse). These models were examined using generalized ordered logit models with the autofit specification which uses an iterative process to identify the partial proportional odds model that best fits the data. When trying ordered logistic regression, the models violated the parallel regression assumption using the Brant test (the models were unweighted for this test as Brant does not support weights). Subsequently, gologit2 was used to estimate models that were less restrictive than the proportional odds/parallel lines models estimated by ordered logistic models (whose assumptions are often violated) but more parsimonious and interpretable than those estimated by a non-ordinal method, such as multinomial logistic regression. The results are reported as odds ratios (ORs). For each abuse outcome, there are two models. The first is without country controls and the second includes countries to account for geographic and cultural differences, among others. All models included the weighting factor for the subset of women who were randomly selected and interviewed in privacy for the domestic violence module. The confidence intervals for all models are given in Appendix A Table A2. All analyses were conducted with Stata 18 statistical software.

## 3. Results

Table 2 reports the percent or mean for each of the abuse, sources of knowledge, and control variables in the analyses for the overall sample and by country. On average, women reported experiencing 1 out of the 7 specified physical abuse encounters compared to scores of 0.6 in The Gambia, 1.2 in Liberia, 0.2 in Senegal, and 1.3 in Sierra Leone. Similarly, women reported scores of 0.6 for experiences of emotional abuse out of the 3 specified compared to 0.4 in The Gambia, 0.8 in Liberia, 0.2 in Senegal, and 0.9 in Sierra Leone. In the overall sample, women reported experiencing scores of 1.8 with regard to the specified control issues in their partnership; 1.3 experienced control issues in The Gambia compared to 2.3 in Liberia, 0.5 in Senegal, and 2.3 in Sierra Leone. For all abuse combined, women reported an average of 3.5 emotional, physical, control, or sexual abuse experiences compared to 2.3 in The Gambia, 4.4 in Liberia, 1 in Senegal, and 4.7 in Sierra Leone. For all four variables of abuse outcomes, Senegal reported the lowest rates and Sierra Leone reported the highest.

Regarding the sources of knowledge, overall, respondents had completed roughly 4.6 years of education, which equates to less than a primary school education. This ranged from a high of 5.48 years in The Gambia to a low of 3.61 years in Senegal. Literacy rates differed across countries. Overall, 28% of women in our sample reported that they could read a sentence compared to 3% in The Gambia, 34% in Liberia, 33% in Senegal, and 22% in Sierra Leone. Concerningly, women in The Gambia had the highest years of completed education and the lowest literacy. Overall, women scored an average of 3 out of 6 for the health knowledge questions, with similar scores across countries.

The controls suggest that overall, 53% of respondents lived in urban areas, were on average 29 years old and had 2.5 children. Overall, 58% of respondents were married compared to 71% in The Gambia, 27% in Liberia, 74% in Senegal, and 64% in Sierra Leone. In the overall sample, 46% of respondents justified beating in at least one scenario compared to 53% in The Gambia, 38% in Liberia, 41% in Senegal, and 50% in Sierra Leone. For wives participating in decision making, in the overall sample, 62% of respondents participated in decision making in at least one scenario compared to 69% in The Gambia, 89% in Liberia, 30% in Senegal, and 58% in Sierra Leone. The respondents reported that their partners had an average of roughly 4.4 years of education with an average of 4.6 in The Gambia, 6.0 in Liberia, 2.4 in Senegal, and 4.4 in Sierra Leone. Lastly, 27% of the sample had at least one other wife in the household compared to 30% in The Gambia, 11% in Liberia, 29% in Senegal, and 32% in Sierra Leone.

**Table 2:** Weighted descriptive table with percent or mean for the overall sample and by country (N = 12,480).

Variables	Overall Category (min, max)	Overall	The Gambia	Liberia	Senegal	Sierra Leone
		% Or Mean (S.E.)	% Or Mean (S.E.)	% Or Mean (S.E.)	% Or Mean (S.E.)	% Or Mean (S.E.)
Abuse Outcomes						
Physical Abuse	(0, 7)	0.95 (0.02)	0.59 (0.03)	1.17 (0.05)	0.20 (0.02)	1.32 (0.03)
Emotional Abuse	(0, 3)	0.63 (0.01)	0.36 (0.02)	0.76 (0.03)	0.15 (0.02)	0.89 (0.02)
Control Issues	(0, 5)	1.80 (0.02)	1.26 (0.04)	2.29 (0.05)	0.48 (0.04)	2.32 (0.03)
Any Abuse	(0, 18)	3.51 (0.04)	2.29 (0.08)	4.38 (0.11)	0.90 (0.07)	4.67 (0.07)
Sources of Knowledge						
Schooling	(0, 25)	4.58 (0.06)	5.48 (0.13)	5.29 (0.15)	3.61 (0.16)	4.34 (0.08)
Literacy	(0, 1)	28.3%	3.1%	33.5%	33.3%	21.9%
Health Knowledge	(0, 6)	3.1 (0.02)	3.7 (0.03)	3.1 (0.04)	3.7 (0.04)	2.6 (0.02)
Controls						
Wealth	(−2.5, 4.07)	0.29 (0.01)	0.34 (0.03)	0.50 (0.03)	0.30 (0.03)	0.13 (0.02)
Urban Residence	(0, 1)	52.8%	72.2%	60.0%	45.9%	41.8%
Married	(0, 1)	58.0%	71.0%	27.0%	73.7%	64.0%
Number of Children	(0, 15)	2.5 (0.02)	2.6 (0.06)	2.4 (0.05)	2.5 (0.07)	2.4 (0.03)
Age	(15, 49)	28.9 (0.11)	28.8 (0.24)	28.8 (0.26)	28.9 (0.28)	28.9 (0.15)
Partner Education	(0, 21)	4.4 (0.08)	4.6 (0.17)	6.0 (0.18)	2.4 (0.18)	4.4 (0.10)
Wife Participating in Decision Making	(0, 1)	61.5%	68.9%	88.5%	29.6%	57.7%
Other Wives	(0, 1)	27.0%	30.1%	11.4%	29.0%	31.5%
Justified Beating	(0, 1)	46.2%	53.0%	38.4%	40.8%	49.6%

S.E. = Linearized standard error in parenthesis when mean is presented.

Table 3 shows the results of the weighted generalized ordered logit regression models of sources of knowledge and reported abuse encounters without (Model 1) and with (Model 2) country. The first abuse outcome looks at women who experienced physical abuse. Looking at the role of schooling, for each step up in education, from no education to one year, for example, women had 1.03 times the odds of experiencing physical violence (*p* = 0.01, OR = 1.03, 95% CI reported in Appendix) in Model 1 without accounting for country and 1.04 (not significant) times the odds of experiencing physical abuse when controlling for country in Model 2. Compared to women who could not read a complete sentence, literacy decreased the odds of physical abuse when not controlling for country (Model 1; *p* < 0.001, OR = 0.62) and when controlling for country (Model 2; *p* = 0.01, OR = 0.75). Similarly, increased health knowledge reduced the odds of experiencing physical abuse in Model 1 (*p* = 0.01, OR = 0.62). Conversely, when country controls were added in Model 2, health knowledge was associated with increased odds of physical abuse (*p* < 0.001, OR = 1.16).

**Table 3:** Weighted generalized ordered logit regression models of sources of knowledge and abuse encounters with and without country (n = 12,480).

	Physical Abuse				Emotional Abuse				Control Issues				Any Abuse			
	Model 1 Model 2				Model 1 Model 2				Model 1 Model 2				Model 1 Model 2			
	OR	S.E.	OR	S.E	OR	S.E.	OR	S.E.	OR	S.E.	OR	S.E	OR	S.E.	OR	S.E.
Sources of knowledge																
Schooling	1.03 **	0.03	1.04	0.03	1.03 **	0.01	1.01	0.01	1.08 ***	0.01	1.03 ***	0.01	1.06 ***	0.01	1.03 ***	0.03
Literacy	0.62 ***	0.06	0.75 **	0.08	0.72 **	0.08	0.90 *	0.11	0.72 **	0.08	1.08	0.10	0.64 ***	0.06	0.92 *	0.08
Health Knowledge	0.62 ***	0.02	1.16 ***	0.03	0.92 ***	0.02	1.15 ***	0.03	0.82 ***	0.02	1.12 ***	0.03	0.85 ***	0.02	1.15 ***	0.03
Controls																
Wealth	0.99	0.04	0.89 *	0.04	1.01	0.05	0.92	0.05	1.05	0.04	0.96	0.04	1.02	0.04	0.92 *	0.04
Urban	1.83	0.06	1.05	0.08	0.84 *	0.06	1.00	0.08	0.84 *	0.06	1.03	0.08	0.82 **	0.05	1.00	0.07
Married	0.15 ***	0.05	0.83	0.08	0.66 ***	0.06	0.94	0.10	0.54 ***	0.04	0.98	0.10	0.53 ***	0.04	0.88	0.08
Number of Children	1.10	0.02	1.04 *	0.02	0.99	0.02	1.01	0.02	0.98	0.01	1.01	0.02	0.99	0.01	1.03	0.02
Age	0.99 *	0.00	0.98 ***	0.00	1.00	0.00	0.99 *	0.00	0.99 **	0.00	0.97 ***	0.00	0.99 **	0.00	0.98 ***	0.00
Wife Decision	1.26 ***	0.07	1.03	0.06	1.30 ***	0.08	1.04	0.07	1.50 ***	0.09	0.95	0.07	1.27 ***	0.06	0.89 *	0.05
Other Wives	0.76	0.07	1.00	0.07	1.05	0.07	1.04	0.07	1.07	0.06	1.11	0.07	1.09	0.06	1.11 *	0.06
Justified Beating	1.95 ***	0.11	1.91 ***	0.11	1.64 ***	0.10	1.62 ***	0.10	1.61 ***	0.08	1.52 ***	0.08	1.78 ***	0.09	1.75 ***	0.09
Husband Educ.	1.03 ***	0.01	1.01 *	0.01	1.02 **	0.01	1.00	0.01	1.03 ***	0.01	1.01	0.01	1.03 ***	0.01	1.01	0.01
Country (The Gambia = ref.)																
Liberia			2.16 ***	0.26			2.77 ***	0.32			3.81 ***	0.47			3.78 ***	0.36
Senegal			0.38 ***	0.05			0.41 ***	0.06			0.26 ***	0.03			0.28 ***	0.03
Sierra Leone			2.18 ***	0.26			3.38 ***	0.30			3.56 ***	0.31			4.77 ***	0.34

S.E. = Linearized standard error; Educ = Education. Model 1 = excluding country, Model 2 = including country. *** *p* ≤ 0.001 ** *p* ≤ 0.01 * *p* ≤ 0.05.

The second abuse outcome is emotional abuse. Increased education was associated with increased odds of emotional abuse without country controls (Model 1; *p* = 0.01, OR = 1.03) and was no longer significant when controlling for country. Conversely, literacy reduced the odds of experiencing emotional abuse for both Model 1 (Model 1; *p* = 0.01, OR = 0.72) and Model 2 (Model 2; *p* = 0.05, OR = 0.90). As with physical abuse, health knowledge was associated with decreased odds of emotional abuse when country controls were excluded from the model (Model 1; *p* < 0.001, OR = 0.92); but when countries were added, health knowledge became related to increased odds of emotional abuse (Model 2; *p* < 0.001, OR = 1.15).

Experiences with both control issues and any abuse followed similar patterns as physical and emotional abuse. Increased education was associated with increased odds of DV for Model 1, but unlike the other models, education remained statistically significant when controls for countries were added in Model 2. Conversely, literacy was associated with decreased odds of abuse in both Models 1 and 2 (excluding Model 2 for control issues). As with physical abuse and emotional abuse, health knowledge was associated with decreased odds of experiencing control issues and any abuse when country controls were not present (Model 1), but was associated with increased odds once country controls were added (Model 2).

With The Gambia being the reference, Senegal had the lowest odds of abuse across the four abuse outcome categories. Sierra Leone had the highest odds across three of the four abuse outcomes (excluding control issues). The controls suggest that having an increased justification for beating is associated with increased odds of physical abuse, emotional abuse, control issues, and any abuse in both Models 1 and 2 and a husband with higher education is associated with increased abuse in Model 1 for physical abuse, emotional abuse, control issues, and any abuse. Conversely, urban residence, being married, and increased age are associated with decreased odds of physical abuse, emotional abuse, control issues, and any abuse. When not controlling for country (Model 1), the results suggest that the respondent participating in decision making is associated with increased odds of the four abuse outcomes. However, when controlling for country (Model 2), the respondent participating in decision making is generally no longer significant or has reduced odds of any abuse.

## 4. Discussion

The three types of education—formal years of schooling, literacy, and health knowledge—had a significant impact on abuse encounters. Furthermore, the results suggest that schooling, literacy, and health knowledge do function as separate entities, with their relationship being context-specific and complex. These findings add to the existing knowledge base of education and domestic violence intersections, providing possible explanations for the differing findings on whether education is a protective factor for DV [7]. Compartmentalizing the conceptualization of “education” into distinct pathways of knowledge has allowed for deeper understanding of what types of learning and skill attainment might ameliorate violence for women in West Africa. Overall, this study demonstrates how nuanced knowledge attainment and education are and how difficult it can be to provide robust solutions to domestic violence rates in West Africa.

This research suggests that literacy has the most reliable beneficial impact on domestic violence as it was the only source of knowledge that was consistently associated with decreased odds of abuse across all abuse encounters and models. Literacy, measured in this study as the ability to read a whole sentence, seems to help women in West Africa decrease their odds of experiencing abuse. It is important to note that literacy is low in all four of these countries with at most 34% being literate in Liberia, compared to a low of 3% in The Gambia. This suggests that if literacy is a protective factor against abuse, there is much progress that can be made through targeting literacy in schools, religious institutions, communities, government policy, etc.

Just as sources of knowledge act in nuanced ways, so do forms of DV. For both physical and emotional abuse, formal schooling was associated with increased violence when excluding country controls and was no longer significant when considering country variation. This is different from control issues where formal schooling remained a risk factor for abuse in both Models 1 and 2. Similarly, literacy is a protective factor for both physical and emotional abuse when including and excluding country controls but is no longer significant when country controls are added for control issues. This suggests that control issues may function differently than physical and emotional abuse and warrant further investigation. While only speculation, perhaps controlling spouses through jealousy, limiting their contact with others, and monitoring their movement is easier to hide or excuse in communities (i.e., higher-educated) that might be more vigilant when looking for signs of “typical” abuse (hitting, threatening, insulting, etc.). Future research should investigate the ways that partner control issues manifest in communities, as control issues were the most frequently experienced form of abuse across all countries in this study and may act in unique ways compared to the more frequently studied forms of abuse.

Further, the results of this study show that broadly, Senegal had the lowest prevalence of domestic violence. Senegal’s higher literacy rates likely had an impact on the lower prevalence of domestic violence. One possible explanation for Senegal’s higher literacy rate could be the governmental and international partner buy-in on working to reduce illiteracy. For example, the Senegalese government is active in promoting non-formal education adult literacy interventions such as the Women’s Literacy Project that offered integrated literacy in local languages [34,35]. Another example of these interventions is the UNESCO Literacy Project for Girls and Women using ICT, which takes advantage of Senegal’s relatively high internet coverage to provide formal and non-formal access to learning [36]. Television, online, and mobile courses were provided in addition to digital kits to physical classrooms to instill literacy skills in girls and women. These interventions, as well as this study, make a case for educational pathways outside of formal schooling to be implemented and researched in West African cultures.

Another finding of this study was that educational attainment in formal schooling was associated with an increased risk of all abuse encounters, agreeing with some of the existing literature [15]. As discussed, advocates and researchers often promote education as an avenue towards decreasing violence against women, but these results suggest that this view is insufficient. A possible explanation for educational attainment being associated with increased DV could be that higher levels of female education may disrupt existing gender norms and power dynamics that expose women to increased DV [37]. Other researchers have demonstrated that equating a woman’s empowerment to solely her education is not only incorrect but also deficient to provide her with freedom from violence [38]. The findings of this study would corroborate this theory, showing that not only is education a multi-faceted experience spanning individual experiences, goals, and definitions, so is women’s empowerment. This finding does not suggest that girls should be removed from school. Rather, it proposes that ensuring these interventions are not exposing women to increased risks of abuse at school and at home should be an individual, community, and governmental task. Future research must be careful to explore the dimensions of education as well as gender equality, noting popular ideas or definitions that might impede our ability to understand the issues at hand.

Finally, the outcomes related to health knowledge demonstrated that the relationships analyzed in this study are context-specific. Overall, health knowledge could be considered as a protective factor, but when controlling for country, health knowledge increased the risk of physical abuse, emotional abuse, control issues, and any abuse. The logic is that in Model 1, countries with higher health literacy have lower abuse overall, but within these countries (Model 2), health literacy has a positive relationship with abuse. Similarly, a wife participating in decision making increased the odds of experiencing the different abuse outcomes when not controlling for country, but when controlling for country, it was shown not to be a risk factor. A possible explanation for this could be the convoluted nature of power in West Africa. While power for women in these countries can lead to better outcomes, men sometimes perceive a woman’s empowerment as a threat to traditional male roles and dominance, resulting in men participating in abuse [7,37].

Moreover, analyzing the separate countries gave insight into how the experience of domestic violence is not universal for women across West Africa. Different countries hold different policies, practices, and ideals which impact how women are viewed, treated, and gain access to different sources of knowledge. These complicated results suggest the need for further research that continues to separate areas of learning in distinct settings to illuminate how cultural beliefs, individual circumstances, and knowledge outcomes interact with both a woman’s empowerment and safety.

The limitations of this study may include the above-mentioned nature of the DHS data sets as well as potential bias in conducting a secondary analysis of survey data. Additionally, we note that only association rather than causation can be identified through our methods and utilization of data. Finally, we note that due to the year of available data for the West Africa region, current world events could have caused cultural shifts and beliefs not accounted for in this study. Despite these limitations, this study provides useful insights into the intersection of domestic violence and education in this region.

## 5. Conclusions

These contextual differences allow for a deeper exploration into reasons why rates of DV may be exacerbated or mitigated. For example, the above-mentioned interventions in Senegal including the Women’s Literacy Project and the UNESCO Literacy Project for Girls and Women using ICT may have been successful due by fostering interactions between the distinct types of knowledge attainment rather than conflating them. These interventions centered reading and writing, but also fostered learning activities on basic skills including health, nutrition, technology, and economics. The Senegalese government declared the Women’s Literacy Project a success and has continued with similar interventions, furthering the quality of literacy programs [34,35]. Likewise, the UNESCO project saw improved basic literacy and vocational skills in girls and women [36]. Similar non-formal education programming has also been prioritized in The Gambia, which had the second-lowest domestic violence prevalence in this study [39,40].

These non-formal education interventions combine different types of knowledge. The example of the Women’s Literacy Project demonstrates this intersection, as literacy and health knowledge were both addressed. Furthermore, the interventions often have the explicit goal of empowering the target population by focusing on marginalized communities, appealing to cultural values, teaching in local languages, and equipping women with skills [34,36]. While non-formal education is a form of educational attainment, it is often omitted from the conversations that researchers and advocates have when simply citing education as a solution. The findings of this study suggest that the common definition of “education” does not encompass the factors that may decrease DV. Limiting research and interventions to just “education” or literacy disregards a large part of the story. This way of thinking does not accurately address what type of education protects women or if a woman’s education results in literacy or knowledge goals, such as health knowledge proficiency or economic understanding. It also disregards whether a woman becomes empowered in her community, her family, and in the decisions revolving around her own health. As seen in the results, the simple measure of years of formal schooling does not necessarily protect women from various forms of abuse.

Schooling, health knowledge, and literacy obviously matter for the wellbeing of women. However, because there are conflicting findings and perspectives on how these pathways of knowledge interact with a woman’s safety, it becomes a matter of ensuring that these interventions are not exposing women to increased risks of abuse. For example, while the results show that the rate of health knowledge is higher when abuse prevalence is lower, when controlling for country, health knowledge is associated with an increased risk of abuse. It is imperative that when increasing the health knowledge of women, researchers and policy implementers make certain that the avenue chosen does not become a risk factor for abuse. One area for future research should be a focus on *why* women can be at risk as they gain more knowledge and how to interrupt this dangerous trend.

Defining education as just schooling ignores educational outcomes like literacy and health knowledge. Furthermore, literacy, schooling, or health knowledge alone do not equate to empowerment. Nelly Stromquist states that “empowerment can succeed only if it is [through] mode[s] of learning [that are] close to the women’s everyday experiences and if it builds upon the intellectual, emotional, and cultural resources the participants bring to their social space” [41]. Future research should explore how to combine the already existing infrastructure of formal schooling with literacy and health knowledge programs that include cultural values. Additional future research and interventions should also work to understand the mechanisms through which knowledge and the nuanced aspects of education put women at greater risk and include continual observation to ensure that these women are safe when utilizing these resources. Future studies should also work towards instilling standardized and distinct ways to discuss aspects of education and should include contextual factors by creating surveys particularly for this research area. Doing so will help policy makers, researchers, nonprofit agencies, and governments address the intricate issues that women in West Africa face and create better avenues towards true education and empowerment.

## Figures and Tables

**Table 1:** Sample size by country.

Country	Full Sample	Surveyed in Domestic Violence Module
The Gambia	14,428	2547
Liberia	9502	2907
Senegal	10,429	1891
Sierra Leone	18,096	5135
Total n	52,455	12,480

DV = Domestic Violence.

## Data Availability

Supporting data can be acquired at dhsprogram.com. URL accessed 10 October 2021.

## Appendix A

**Table A1:** Full list of survey questions.

Construct	Survey Questions
Physical Abuse	Have you never, often, sometimes, or yes but not in the last 12 months… - been pushed, shook or had something thrown by husband/partner - been slapped by husband/partner - been punched with fist or hit by something harmful by husband/partner - been kicked or dragged by husband/partner - been strangled or burnt by husband/partner - been threatened with knife/gun or other weapon by husband/partner - had arm twisted or hair pulled by husband/partner
Emotional Abuse	Have you never, often, sometimes, or yes but not in the last 12 months… - Ever been humiliated by husband/partner - Ever been threatened with harm by husband/partner - Ever been insulted or made to feel bad by husband/partner
Control Issues	Yes or no… - Husband/partner jealous if respondent talks with other men - Husband/partner accuses respondent of unfaithfulness - Husband/partner does not permit respondent to meet female friends - Husband/partner tries to limit respondent’s contact with family - Husband/partner insists on knowing where respondent is
Sexual Abuse	Have you never, often, sometimes, or yes but not in the last 12 months… - Ever been physically forced into unwanted sex by husband/partner - Ever been forced into other unwanted sexual acts by husband/partner - Ever been physically forced to perform sexual acts respondent didn’t want to

**Table A2:** The 95% confidence intervals for Table 3.

	Physical Abuse				Emotional Abuse				Control Issues				Any Abuse			
	Model 1 Model 2				Model 1 Model 2				Model 1 Model 2				Model 1 Model 2			
	95% Conf. Interval				95% Conf. Interval				95% Conf. Interval				95% Conf. Interval			
Sources of knowledge																
Schooling	1.01	1.05	1.00	1.06	1.01	1.05	0.99	1.03	1.05	1.10	1.01	1.05	1.04	1.07	1.01	1.05
Literacy	0.50	0.76	0.60	0.79	0.58	0.89	0.71	1.14	0.58	0.90	0.89	1.30	0.53	0.76	0.75	1.09
Health Knowledge	0.41	0.94	1.03	1.43	0.88	0.97	1.08	1.22	0.79	0.86	1.06	1.19	0.82	0.89	1.10	1.21
Controls																
Wealth	0.90	1.07	0.90	1.11	0.92	1.10	0.84	1.02	0.97	1.14	0.88	1.05	0.95	1.11	0.85	1.00
Urban	0.70	4.77	0.80	1.14	0.72	0.98	0.85	1.17	0.73	0.96	0.89	1.20	0.72	0.94	0.88	1.15
Married	0.07	0.34	0.69	1.09	0.56	0.78	0.76	1.17	0.46	0.62	0.80	1.18	0.46	0.62	0.73	1.06
Number of children	0.93	1.31	1.00	1.08	0.96	1.03	0.98	1.05	0.95	1.01	0.97	1.04	0.96	1.02	0.99	1.06
Age	0.98	1.00	0.97	0.99	0.99	1.01	0.98	1.00	0.98	1.00	0.97	0.98	0.98	1.00	0.97	0.98
Wife decision	1.13	1.41	0.95	1.24	1.16	1.46	0.91	1.18	1.33	1.69	0.82	1.10	1.16	1.40	0.80	0.99
Other Wife	0.20	2.82	0.93	1.24	0.92	1.19	0.92	1.19	0.96	1.19	0.99	1.25	0.98	1.21	1.00	1.25
Justify Beating	1.74	2.18	1.60	2.08	1.46	1.84	1.44	1.82	1.46	1.77	1.37	1.68	1.62	1.96	1.59	1.93
Husband Educ.	1.02	1.05	0.99	1.02	1.01	1.03	0.99	1.01	1.02	1.04	0.99	1.02	1.02	1.04	1.00	1.02
Country (The Gambia = ref.)																
Liberia			1.38	2.18			2.22	3.47			3.00	4.85			3.14	4.56
Senegal			0.27	0.46			0.31	0.53			0.21	0.33			0.23	0.33
Sierra Leone			1.96	2.76			2.83	4.03			3.00	4.23			4.15	5.50
